# Systematic estimation of cystic fibrosis prevalence in Chinese and genetic spectrum comparison to Caucasians

**DOI:** 10.1186/s13023-022-02279-9

**Published:** 2022-03-21

**Authors:** Qi Ni, Xiang Chen, Ping Zhang, Lin Yang, Yulan Lu, Feifan Xiao, Bingbing Wu, Huijun Wang, Wenhao Zhou, Xinran Dong

**Affiliations:** 1 Children’s Hospital and Institutes of Biomedical Sciences, Fudan University, National Children’s Medical Center, Shanghai, 201102 People’s Republic of China; 2 Center for Molecular Medicine, Children’s Hospital of Fudan University, National Children’s Medical Center, Shanghai, People’s Republic of China; 3 Division of Neonatology, Children’s Hospital of Fudan University, National Children’s Medical Center, Shanghai, People’s Republic of China; 4 Department of Endocrinology and Inherited Metabolic Diseases, Children’s Hospital of Fudan University, Shanghai, People’s Republic of China

**Keywords:** Cystic fibrosis, Screening panel, Prevalence estimation, Allele frequency comparison, Haplotype construction and comparison

## Abstract

**Background:**

Cystic fibrosis (CF) is a common, life-threatening genetic disease in Caucasians but rarely reported in Chinese population. The prevalence and population-specific genetic spectrum of CF in China needs to be systematically estimated and compared with Caucasians.

**Materials and methods:**

We reviewed 30,951 exome-sequencing samples, including 20,909 pediatric patient samples and 10,042 parent samples, from Chinese Children's Rare Disease Genetic Testing Clinical Collaboration System (CCGT). After the in-lab filtration process, 477 candidate variants of *CFTR* gene were left and 53 variants were manually curated as pathogenic/likely-pathogenic (P/LP). These P/LP variants were adopted to estimate CF prevalence in three methods: the carrier frequency method, the permutation-combinations method and the Bayesian framework method. Allele frequencies of the 477 *CFTR* variants were compared with non-Finland European (NFE) and East Asian (EAS) from gnomAD database. To investigate the haplotype structure difference of *CFTR*, another 2067 whole-genome-sequencing samples from CCGT and 195 NFE from 1000 genome project were analyzed by Shapeit4 software.

**Result:**

With the 53 manually curated P/LP variants in *CFTR* gene, we excluded individuals identified or suspected with CF and their parents in our cohorts and estimated the Chinese CF prevalence is approximately 1/128,434. Only 21 (39.6%) of the 53 variants were included in Caucasian specific CF screening panels, resulting in significantly under-estimation of CF prevalence in our children cohort (1/143,171 vs. 1/1,387,395, *P* = 5e−24) and parent’s cohort (1/110,127 vs. 1/872,437, *P* = 7e−10). The allele frequencies of six pathogenic variants (G970D, D979A, M469V, G622D, L88X, 1898+5G->T) were significantly higher in our cohorts compared with gnomAD-NFE population (all *P*-value < 0.1). Haplotype analysis showed more haplotype diversity in Chinese compared to Caucasians. In addition, G970D and F508del were founder mutation of Chinese and Caucasians with two SNPs (rs213950-rs1042077) identified as related genotype in exon region.

**Conclusions:**

Chinese population showed significantly different genetic spectrum pattern in *CFTR* gene compared with Caucasian population, and thus a Chinese-specific CF screening panel is needed.

**Supplementary Information:**

The online version contains supplementary material available at 10.1186/s13023-022-02279-9.

## Introduction

Cystic fibrosis (CF) is an inherited autosomal recessive disease that threatens the patients’ whole life. Previous studies found that CF is more common in Caucasian population than in other populations [1]. The preference of CF is approximately 1 in 3000 for Caucasians, 1 in 4000–10,000 for Latin Americans and 1 in 15,000–20,000 for African Americans [2, 3]. In the United States, CF occurs in approximately 1 in 4000 newborns [4]. However, the reported CF prevalence is always much lower in Asian countries despite that it varies widely from 1:2560 to 1:350,000 live births [5–7].

The epidemiology of CF has not been well studied in Chinese population. Most published studies focused on the genetic and clinical characteristics of CF in Chinese patient populations. Chinese CF patients have been shown to have novel and different frequencies of the CF transmembrane conductance regulator (*CFTR*) gene variants, which suggests that CF in Chinese population may have a different spectrum of variants comparing with Caucasian population [8, 9]. For example, G970D (c.2909G>A) was reported as a hot spot in Chinese population while it was not common in Caucasian population and not included in Caucasian screening panels [8]. Therefore, CF screening panels for Caucasian population might not be suitable for Chinese population. What’s more, because newborns in China are not screened for CF, potential patients with CF are not systematically identified and CF may be underreported in China.

In this study, we retrospectively analyzed next-generation-sequencing samples in the Chinese Children's Rare Disease Genetic Testing Clinical Collaboration System (CCGT), which is one of the largest genetic databases of the Chinese pediatric population [10]. Then we applied three different methods to estimate CF prevalence in Chinese population and presented quantitative evidence of how Caucasian CF screening panel is not suitable for Chinese. Furthermore, we systematically compared allele frequencies and haplotype structures between Chinese and Caucasian populations to demonstrate the genetic spectrum differences. Based on these results, we established the panel of CF genetic screening and diagnosis for Chinese population and explained the differences of *CFTR* gene characteristics between Chinese and Caucasian populations.

## Results

### Estimated CF prevalence of Chinese population is lower than Caucasian population

**Fig. 1:**
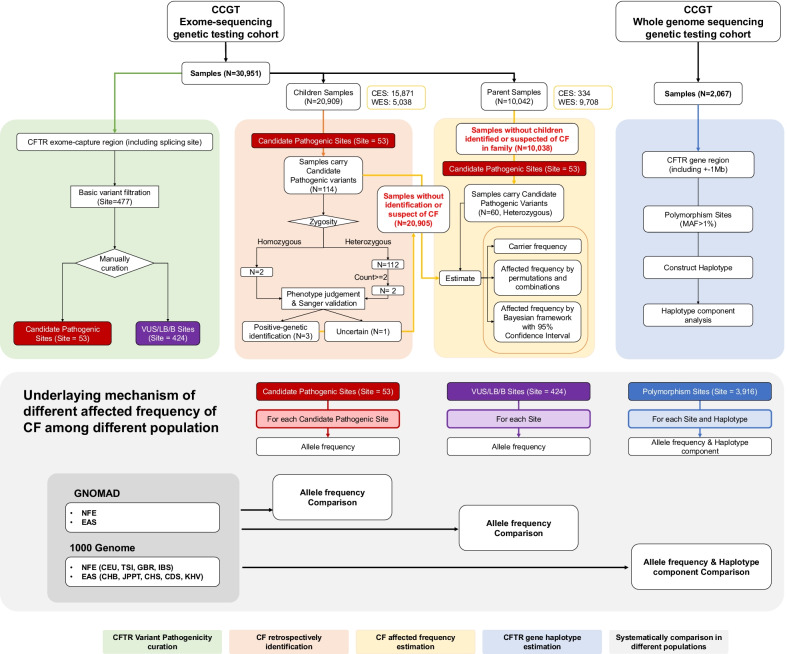
Overall Study design. This study consisted of four parts, and within each part, the systematically comparisons among our two cohorts (representing Chinese population) with public reported cohorts from gnomAD-NFE, 1000genome-NFE (representing Caucasian population) and gnomAD-EAS, 1000genome-EAS (control for east Asian population) were performed: (1) *CFTR* variant pathogenicity curation. (2) CF retrospectively identification. (3) CF affected frequency estimation. (4) *CFTR* gene haplotype estimation

Totally, we enrolled 20,909 pediatric patients as children cohort and 10,042 parental samples as parent’s cohort (Fig. 1). After filtration and manually quality assessment for *CFTR* variants in this two cohorts, 53 P/LP variants were identified (Additional file 1: Table S1). To estimate CF prevalence, we excluded children identified or suspected with CF and their parents, left 20,905 children and 10,038 parents. In the children cohort, the affected frequency of CF was ranged from 1/153,825 to 1/143,171. In the parent’s cohort, the estimated CF frequency was ranged from 1/120,528 to 1/110,127 (Table 1). The average estimated prevalence of Chinese CF was around 1/128,434, much lower than in Caucasians (1 in 3000) and other populations (Latin Americans: 1 in 4000–10,000, African Americans: 1 in 15,000–20,000) [2, 3].

**Table 1:** Children cohort and parent’s cohort with estimated affected frequency by three methods

	Children cohort	Parents cohort
Total number	20,905	10,038
Gender (Male/female)	12,773/8132	5012/5026
CES/WES	15,869/5036	334/9704
Heterozygous of P/LP variants	110 (73 males, 37 females)	60 (38 males, 22 females)
Carrier frequency	1/190	1/167
Couple’s carrier risk	1/36,117	1/27,989
Method 1: estimated affected frequency by carrier frequency	1/144,469	1/111,957
Method 2: estimated affected frequency by permutation-and-combination	1/153,825	1/120,528
Method 3: estimated affected frequency by Bayesian framework (95% confidence interval)	1/143,171 (1/213,769–1/101,160)	1/110,127 (1/192,111–1/69,638)
Average estimated prevalence (mean value of the above six frequencies)	1/128,434	

### CF screening panels for Caucasians underestimate CF prevalence in Chinese

We treated the identified 53 P/LP variants as a Chinese-specific CF screening panel. Based on this panel, we retrospectively identified three CF patients (Additional file 2: Figure S1). Patient 1 was a 10-year-old boy with two compound heterozygous pathogenic variants F312del (c.935_937delTCT) and 2184insA (c.2052dupA). Both variants were annotated as DM in HGMD. F312del was inherited from the patient’s mother, and 2184insA was a de novo variant. Patient 1 was diagnosed as CF with clinical phenotypes of hepatic cirrhosis and hepatosplenomegaly. Patient 2 was a 12-year-old boy diagnosed with hepatosplenomegaly and increased serum hepatic transaminase. A homozygous splicing variant in intron5 711+4TG->CA (c.579+4_579+5delTGinsCA) of patient 2 was identified by CES. This rare variant was predicted to have a high risk of leading to a broken site and subsequently resulting in erroneous mature mRNA constitution according to the Human Splicing Finder matrices [11] and MaxEnt algorithms [12]. Sanger sequencing found that the homozygous splicing variant was inherited separately from his parents. Both two patients had a negative family history of CF. Patient 3 was an 11-year-old girl with bronchiectasis and recurrent pneumonia. *Pseudomonas aeruginosa* was found in the sputum culture test. A homozygous stop-gained variant L88X (c.263T>G) was detected in patient 3 by WES and confirmed by Sanger sequencing that the homozygous variant was inherited from the patient’s parents. Besides, we identified another patient carrying two compound heterozygous pathogenic variants 1291delTT (c.1159_1160delTT) and 1380ins7 (c.1242_1243insAACAAAC) without any typical phenotypes, waiting for follow-up interview.

**Fig. 2:**
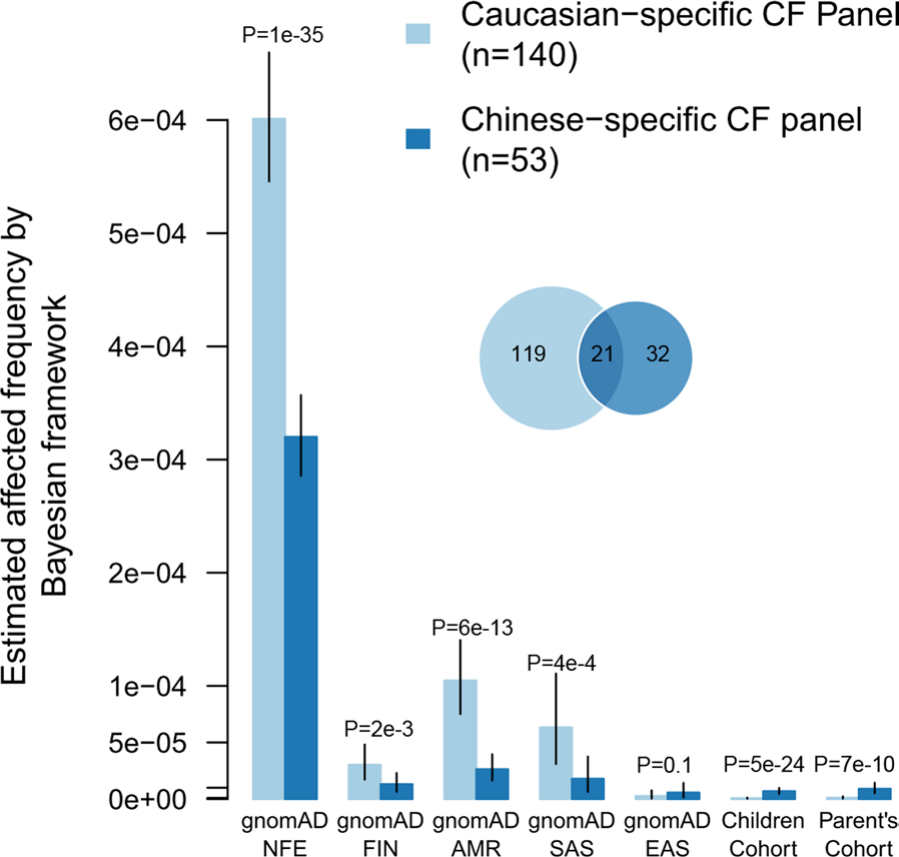
Estimated affected frequency on two screening panels (Caucasian, Chinese) by Bayesian framework. The Venn-graph shows the screening variants intersection between Chinese-specific panel and Caucasian-specific panel. Each cohort has two bars with each showing the estimated affected frequency if only use the pathogenic variants in the panel, and the *P*-value above shows the significance for difference. The vertical line shows the 95% confidence interval

We reviewed typical CF screening panels applied in Caucasians and summarized 140 *CFTR* variants as a Caucasian-specific CF screening panel (Additional file 3: Table S2). We compared the Caucasian-specific CF screening panel and the Chinese-specific panel, and found only 21 variants was shared, which indicated the distinct genetic background in the two populations. Then we applied these two CF screening panels to estimated CF affected frequency in our cohorts and other populations from gnomAD database with Bayesian framework method (Fig. 2 and Additional file 2: Table S3). The results showed that Caucasian–specific CF screening panel detected much higher affected frequencies in NFE, FIN, AMR and SAS populations, and lower in EAS and our two Chinese cohorts (all *P* < 0.1). Meanwhile the Chinese-specific CF screening panel detected higher affected frequencies in EAS and our two cohorts than in other populations. Notably, CF prevalence would be significantly underestimated in both Chinese children cohort (OR = 9.69, from 1/143,171 to 1/1,387,395, *P* = 5e−24) and parent’s cohort (OR = 7.92, from 1/110,127 to 1/872,437, *P* = 7e−10) if using the Caucasian-specific screening panel.

### Allele frequencies of *CFTR* variants in Chinese is distinct from Caucasians

**Fig. 3:**
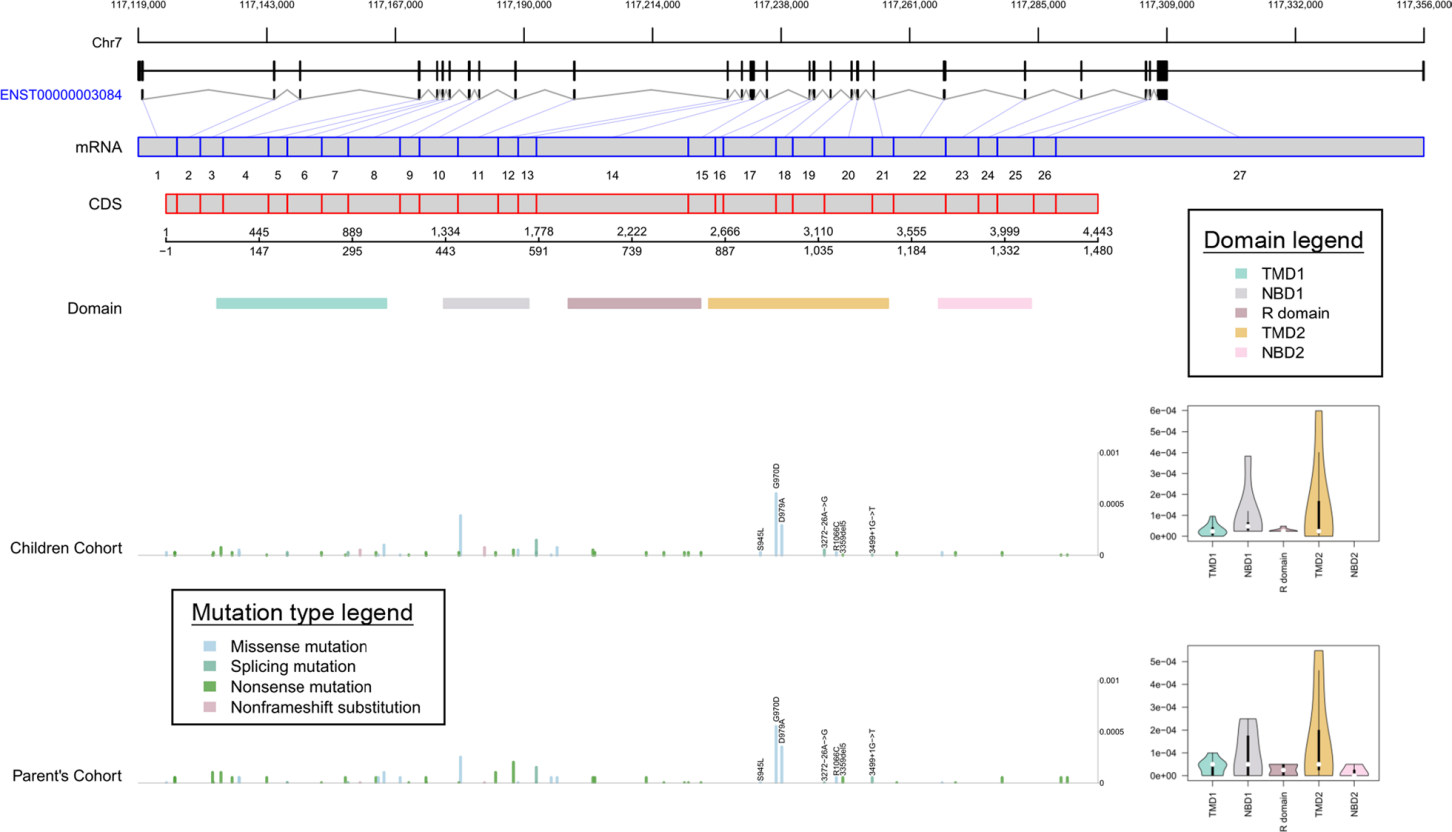
Allele frequencies of P/LP variants in different domains of CFTR protein. P/LP variants are mapped to CFTR protein domains. Different color label different domains. The height of each bar represents the allele frequency of each variant in each cohort. The violin plot summarizes the total allele frequency of pathogenic variants in each domain

To further detect the *CFTR* genetic differences between Chinese and Caucasians, we mapped the 53 P/LP variants to CFTR protein structure and calculated variants allele frequencies (AF) of each protein domain. Seven out of 36 protein-related P/LP variants were located in transmembrane domain 2 (TMD2). The total AF of these 7 variants in our children cohort was 1.96 $\times$ 10^–3^ and 2.09 $\times$ 10^–3^ in parent’s cohort, which was higher than the AF in other four domains (Fig. 3, OR ≥ 1.5), while most variants in Caucasians located in NBD1 domain (Additional file 2: Figure S2). Besides, top two frequent P/LP variants (G970D and D979A) were both located in TMD2 in our Chinese cohorts. These results indicate that TMD2 may be the most important disease-related domain for Chinese population. We also compared the AF of the P/LP variants for four mutation types in different populations (Additional file 2: Figure S3). Four missense variants (G970D, D979A, M469V, G622D), one nonsense variant (L88X) and one splicing variant (1898+5G->T) had significant higher AF in our two cohorts than in gnomAD-NFE (all *P* < 0.1). Two missense variants (R117C, R117H) and one non-frameshift substitution (F508del) had significantly lower AF in our population (all *P* < 0.1).

From the 424 variants curated as VUS or benign in our cohorts, 116 variants were reported as DM in HGMD or P/LP in ClinVar database. After comparing the AF of these conflicting variants among children cohort, parent’s cohort, gnomAD-EAS, and gnomAD-NFE, we found that nineteen variants (I125T, N186K, E217G, N287K, 1342-6T−>A, K411E, S485C, I556V, F650L, E681V, T760M, 2752-97C->T, S895N, R1070Q, R1097C, 3791C/T, 3849+45G->A, Q1352H, R1453W) had significantly higher AF (all *P* < 4e−7 and OR > 10) in our children and parent’s cohorts (Additional file 2: Figure S4). For example, Q1352H has AF $6.5\times$ 10^–3^ in our children cohort and $6.4\times$ 10^–3^ in our parent’s cohort compared with no-reporting in gnomAD-NFE (*P* < 1e−100 and OR = Inf).

In addition, we compared the AF of polymorphism sites around and on *CFTR* gene (± 1 Mb region) with 2067 CCGT-WGS cohort and gnomAD-NFE, as most SNPs located out of the capture region of exome-sequencing kits. Seventy-eight intron-SNPs had higher minor allele frequency (MAF) in CCGT-WGS cohort, while ten intron-SNPs and two linked exon-SNPs, 4389G/A (c.4389G>A, rs1800136, AF = 0.235) and chr7:117308413:C>T (c.*1251C>T, rs1042180, AF = 0.013), had significantly higher MAF in gnomAD-NFE. The SNPs pattern of CCGT was different from NFE, but similar with EAS (Additional file 2: Figure S5).

### Haplotype analysis indicated more haplotype diversity in Chinese population

**Fig. 4:**
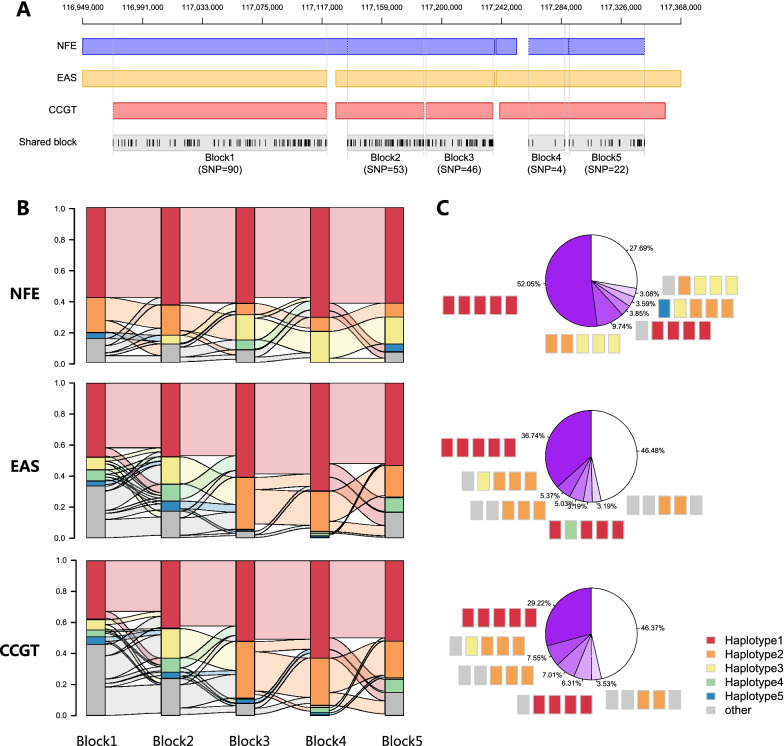
Haplotype comparison between CCGT, EAS and NFE. **A** Haplotype structure for 1000genome-NFE, 1000genome-EAS and CCGT-WGS cohorts. The shared blocks with tagged SNPs (vertical line) are the intersected regions of these three cohorts. Only haplotype blocks with length larger than 10 kb were remained. Five haplotype blocks in 1000genome-NFE, three blocks in 1000genome-EAS and four blocks in CCGT-WGS cohorts were found, resulting in five shared blocks. **B** The distribution of haplotype frequency for each shared block in three cohorts. For each shared block, top five high frequency haplotypes were shown in color and the rest were combined as “other” in grey. The Sankey ribbon between each of the adjacent blocks showed the haplotype intersection statistics. For example, in 1000genome-NFE, 100% of the top 1 haplotype in the shared block 1 were accompanied by top 1 haplotype in the shared block 2. **C** Pie chart for the haplotype frequencies in the three cohorts. The colored chain rectangles indicated the combined haplotype construction of the five shared blocks for the adjacent sector

To explore the underlying mechanism of different *CFTR* genetic spectrum between Chinese and Caucasian populations, we analyzed the haplotype pattern based on WGS data. As gnomAD does not provide individual genotype data, we applied the 2067 WGS cohort from CCGT for haplotype structure construction compared with 195 NFE and 298 EAS from 1000 genome WGS database. Among the three WGS cohorts, five shared haplotype blocks were detected (Fig. 4A). The haplotype construction of the five shared blocks were significantly different between 1000genome-NFE and 1000genome-EAS (all *P* < 5e−16), and different between 1000genome-NFE and CCGT-WGS cohort (all *P* < 5e−20), while 1000genome-EAS and CCGT-WGS were only significantly different in the first three blocks (all *P* < 1e−4) (Fig. 4B). When combining the five blocks together, the most frequent haplotype consists 52.05% of 1000genome-NFE, much higher than 36.47% for 1000genome-EAS and 29.22% for CCGT-WGS (all *P* < 3e−3), indicating less *CFTR* haplotype diversity in Caucasians compared with Chinese (Fig. 4C).

### Different founder mutations and founder genotypes of *CFTR* were detected in Chinese and Caucasian population

**Fig. 5:**
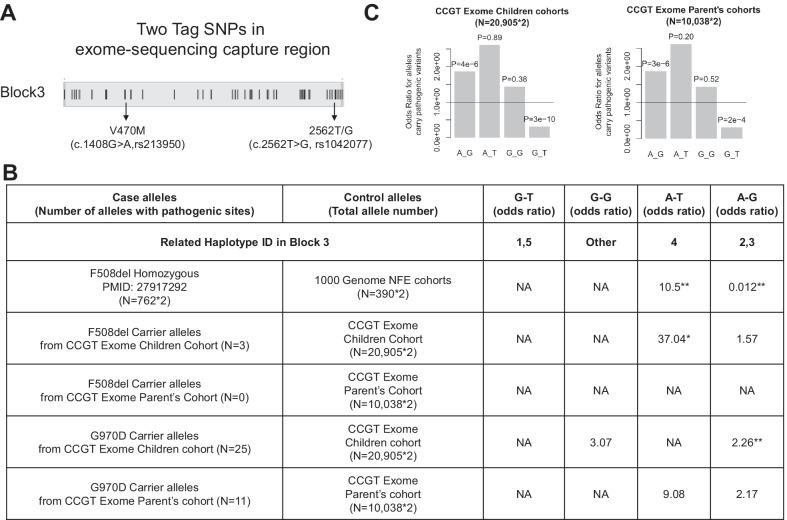
Joint genotype analysis for *CFTR* gene. **A** Location of two exon-tag SNPs (rs213950, rs1042077) in the shared haplotype block 3. **B** Odds ratio of allele frequencies for different rs213950-rs1042077 joint genotype (column) in each cohort (row). **C** Odds ratio of allele frequencies for different rs213950-rs1042077 joint genotype for alleles carry 53 pathogenic variants in CCGT exome sequencing cohorts

There were two exon SNPs located in haplotype block 3, V470M (c.1408G>A, rs213950) and 2562T/G (c.2562T>G, rs1042077) (Fig. 5A), allowing us to study the exon-only joint genotype with P/LP variants in our large-scale children and parent’s exome-sequencing cohorts. Previous study had reported the *CFTR* variant F508del was strongly related with the joint genotype “A-T” (combination of rs213950-rs1042077) [13]. The frequency of “A-T” genotype in F508del CF patients was much higher than in 1000genome-NFE (OR = 10.5, *P* = 3.7e−121, Fig. 5B). This finding was consistent in our CCGT exome sequencing cohorts which had 3 F508del carriers in children cohort (OR = 37.04, *P* = 0.04). Furthermore, genotype “A-G” was strongly associated with G970D, which was the most frequent pathogenic variant in our exome-sequencing cohorts (OR = 2.26 with *P* = 4e−3 in children cohort, OR = 2.17 with *P* = 0.09 in parent’s cohort, Fig. 5B). When taking all the 53 P/LP variants together, genotype “A-G” was significantly over-represented in alleles carrying P/LP variants (*P* = 4e−6 in children cohort, *P* = 3e−6 in parent’s cohort, Fig. 5C).

The AF of F508del associated genotype “A-T” is 0.095 in 1000genome-NFE cohort but no more than 0.010 in neither CCGT exome sequencing cohorts, CCGT-WGS cohort nor 1000genome-EAS cohort (Additional file 2: Table S4). The high frequency of genotype “A-T” is consistent with the high frequency of F508del variant in Caucasian population. On the contrary, the AF of G970D associated genotype “A-G” is only 0.269 in 1000genome-NFE cohort but 0.425 in CCGT exome sequencing children cohort, 0.419 in CCGT exome sequencing parent’s cohort, 0.414 in CCGT-WGS cohort, and 0.364 in 1000genome-EAS cohort. In general, F508del and G970D could be founder mutations in Caucasian and Chinese, while genotype “A-T” and “A-G” of rs213950-rs1042077 could be potential risk genotype for *CFTR* P/LP variants in the two populations respectively.

## Discussion

In this study, we provided the estimated prevalence of cystic fibrosis in Chinese population based on a Chinese-specific CF screening panel consisting of manually curated *CFTR* pathogenic or likely pathogenic variants in a large-scale exome sequencing Chinese cohort. We also compared the allele frequencies of pathogenic variants, rare non-pathogenic variants and SNPs between Chinese and Caucasian population to investigate the genetic background difference. We attempted to explain the different *CFTR* genetic spectrum in Chinese and Caucasian by analyzing haplotype structures and detecting founder variants.

The prevalence of CF in Caucasian is reported between 1:3000 and 1:20,000 [2–4], while the CF incidence of Asia population is already known to be much lower than the Caucasian population as 1:2560 to 1:350,000 [5–8]. In this study, we estimated the prevalence in a robust way. Firstly, all variants of *CFTR* were curated by three experienced geneticists. Secondly, we estimated the prevalence in a large-scale cohort where the patients were from across the country and had various phenotypes. Finally, the prevalence of CF was estimated by three methods. The results calculated by the three methods were similar: the Chinese CF prevalence is ranged from 1/153,825 to 1/110,127. Although CCGT were based on patients’ cohort, it is one of the largest genetic databases (*N* = 30,951) that could be used to calculated the rare disease prevalence. Besides, as CF is extremely rare in Chinese population, the patient-based cohort was more likely to present pathogenic variants. So, we used this population to present a relatively high CF prevalence which could benefit the screening and call for physicians’ attention. However, as CCGT is not a naturally gathered healthy individual cohort, the genotype frequency of this population does not correspond with the Hardy–Weinberg equilibration. More accurate and robust CF prevalence could be estimated with a naturally gathered healthy Chinese population.

Because CF has not been included in the Newborn Screening Plan in China, and there were CF patients clinically diagnosed without *CFTR* mutations [14], the Chinese CF patient population may be underestimated. Nowadays, Chinese CF patients’ genetic diagnosis is based on reported *CFTR* variants, most of which are reported in Caucasian population. The Chinese specific variants are unknown. In this study, we recommend the 53 P/LP variants as CF screening panel for Chinese population, especially the six variants with high AF: G970D (c.2909G>A), D979A (c.2936A>C), M469V (c.1405A>G), G622D (c.1865G>A), L88X (c.263T>G), and 1898+5G->T (c.1766+5G>T), which could also be used in clinical diagnosis process. We statistically found that the Chinese CF prevalence would be 10% lower if estimated by Caucasian specific CF screening panel. So, it is essential and inevitable to introduce a Chinese specific CF panel in clinical practice. Though we could not directly provide a definite prevalence value by systematically newborn screening, the population-based statistical prevalence may give a preliminary evidence for the underestimation of CF in Chinese populations.

We described the different characteristics of the *CFTR* gene between Chinese population and Caucasian population. Firstly, the pathogenic variants were enriched in TMDs rather than NBDs in Chinese population, where functions were relative less reported except for drug binding variants [15]. This could partially explain the difference of clinical manifestation of CF between Chinese and Caucasian patients. The three genetic-diagnosed patients in our study had different phenotypes from previously reported patients with the same disease-causing variants [16–18], making genotype–phenotype matching more complicated. Thus, much more patients from different populations are required to draw solid conclusions about genotype–phenotype matching pattern. Secondly, 116 variants, which had been reported as DM in HGMD or P/LP in ClinVar, were curated as VUS or benign level in our study as they had significant higher allele frequencies in our two cohorts. This finding is consistent with previous study. For example, the allele frequency of I556V (c.1666A>G) in Asia population is as high as 4.7% [19], the same AF has been observed in our cohorts (Additional file 1: Table S1). This uncovers the different allele frequency and incomplete penetrance among different populations. Thirdly, polymorphism sites showed the haplotype structure and content were substantial different between Chinese and Caucasians. The frequency of the most frequent haplotype in Caucasian population (60–70%) was much higher than in EAS and in CCGT population (40–50%). The top 5 haplotype combinations accounted for 72% of all haplotypes in Caucasian population, while accounts for 54% in EAS and CCGT population. These demonstrate the lack of haplotype diversity in Caucasian population than in EAS and CCGT population.

Founder mutation could help to explain the lower diversity of haplotype and the high frequency of a certain rare genetic disease in a certain population [20]. Although Chinese and Caucasian populations are large and not isolated, the differences of genetic characteristics still suggest the existence of founder effect. Several studies have reported different founder variants of *CFTR* in various races. F508del was reported to account for 30% to 88% *CFTR* pathogenic variants in non-Chinese populations [19]. Besides, Pompei et al. found that most variants were associated with the M470V (named V470M in our study) allele in several European populations which can help to trace the origin of the V allele [21]. Leung et al. reported a founder variant I1023R (c.3068T>G) in southern Chinese populations [22]. In this study, we curated the I1023R variant as VUS according to ACMG guideline. However, we found another variant G970D with the highest allele frequency (36 samples, accounts for 21.2% carriers) could be a founder variant in Chinese population, which consisted with a previous study [23]. Our results would be more solid with more next generation sequencing data of Caucasian CF patients and Chinese CF patients. More accurate risk haplotypes could be found if large-scale individual whole genome sequencing dataset, especially from patient samples, could be available in future.

## Conclusions

Out study indicated that the genetic spectrum pattern of *CFTR* gene in Chinese population is significantly distinct from Caucasian population, and thus a Chinese-specific CF screening panel is needed.

## Materials and methods

### Collection of Chinese population data

This study was approved by the ethics committees of Children's Hospital of Fudan University (2014-107 and 2015-130). Children and parent’s cohort of CCGT database who underwent genetic tests from December 2015 to December 2019 were all included. The children cohort were those who had the potential of genetic diseases. The parent’s cohort were patients’ healthy parents. Counselling was performed by physicians prior to genetic testing. Informed consents were obtained from the parents of patients. In total, a cohort consisted of 16,205 clinical exome sequencing (CES) data and 14,746 whole exome sequencing (WES) data was used for prevalence estimation. CES was performed using the Agilent ClearSeq Inherited Disease Kit. WES was conducted by the Agilent Sureselect All Exons Human V5 Kit. Both tests run on the Illumina HiSeq X10 with 150 bp pair-end sequencing. Another cohort consisted of 2067 unrelated individuals without CF patients from CCGT who underwent whole genome sequencing (WGS) was used for SNP allele frequency comparison and haplotype estimation (full database was not published, partial samples could be found in [24, 25]). WGS was operated using a Clinical Laboratory Improvement Amendments and sequenced on an Illumina NovaSeq 6000 platform with 150 bp pair-end read length. All kits covered the *CFTR* gene region. The designed capture region on *CFTR* of CES and WES were showed in Additional file 4: Table S5. Quality control steps were showed in Additional file 2: Figure S6. Details of the sequencing and analysis can be found in our previously published papers [24, 26, 27].

### Collection of Caucasian and other populations data

Variant lists of CF screening panel in Caucasian population were collected from public clinical tests and articles (Additional file 3: Table S2). The allele frequencies (AF) of *CFTR* gene in other populations were downloaded from the gnomAD database (V3.1.2) [28]. Non-Finland European, Finnish in Finland, Admixed American, South Asian and East Asian population in gnomAD were used. Gene annotation was from GENCODE [29] (ENSG00000001626, ENST00000003084) and protein domain information was obtained from pfam [30] (uniprot ID: P13569).

Individual genotype datasets from 1000 genome were downloaded from web site [31, 32]. 1000genome-NFE (non-Finland European population) was the combination of CEU (Utah Residents with Northern and Western European Ancestry), TSI (Toscani in Italia), GBR (British in England and Scotland) and IBS (Iberian Population in Spain). 1000genome-EAS (East Asian population) was the combination of CHB (Han Chinese in Beijing, China), JPT (Japanese in Tokyo, Japan), CHS (Southern Han Chinese), CDX (Chinese Dai in Xishuangbanna, China) and KHV (Kinh in Ho Chi Minh City, Vietnam).

### Curation of *CFTR* pathogenic variants

After quality control of sequencing data and in-lab automated filtration process [26], 477 *CFTR* variants were detected. Indel variants were manually checked for HGVS nomenclatures. All variants were mapped to CFTR2 [33] and CFTR1 [34] databases for legacy name. If one variant was not recorded in neither database, a legacy name would be given according to the mutation nomenclature in practice [35]. These *CFTR* variants were curated by three clinical geneticists back-to-back according to the ACMG guideline[36] and CFTR2 database for pathogenicity of CF. After manually curation, in total 53 *CFTR* variants were identified as P/LP variants (Additional file 1: Table S1). The identification of CF patients were made by pulmonary physicians and geneticists together according to a published article [37].

### Estimation of CF prevalence

We divided the 30,951 samples into two sub cohorts: the children cohort (15,871 CES samples and 5038 WES samples) and the parent’s cohort (334 CES samples and 9708 WES samples). To estimate CF prevalence, we excluded samples diagnosed or suspected with CF and their parents, resulting in 20,905 children and 10,038 parents. We obtained the genotype of each 53 P/LP *CFTR* variants in each cohort and estimated CF affected frequency by three methods. The first one was directly based on carrier frequency. The risk for a CF child was defined as the couple’s carrier risk (product of carrier frequency) divided by 4 (for autosomal recessive inheritance model). The second one was based on permutation-and-combination. In this strategy, individual gender was involved in the possibility calculation. The third one was based on Bayesian framework, referred from Schrodi et al. [38], where 95% confidence interval could be estimated. The main step for this strategy was to calculate the allele number with at least one of the P/LP variants and the total allele number in the cohort. The third strategy was also adopted to estimate CF prevalence in other populations with gnomAD allele count dataset. When using gnomAD allele counts, we hypothesized that no sample could have more than one pathogenic variant in *CFTR* gene, which was acceptable for a cohort with disease-free samples. Detailed calculation process of the three methods were described in Additional file 2: Supplementary Notes.

### Estimation of *CFTR* gene haplotypes

Three cohorts, 2067 WGS samples from the CCGT database, 195 NFE samples and 298 EAS samples from 1000 genome database (v5.20130502) were collected. For each cohort, variants information files of *CFTR* (hg19, + − 1 Mb) were extracted and merged. For the CCGT WGS cohort, phasing was processed by shapeit4 [39]. Then phased variant information files of these three cohorts were transformed into plink format. Only single nucleotide polymorphism (SNP) variants with high allele frequency (MAF ≥ 0.01) and passed the Hardy–Weinberg equilibrium exact test (hwe ≥ 0.001) were used. The haplotype block for each population was calculated by using option –blocks 'no-pheno-req'. Finally, we performed haplotype-association test between alleles carrying pathogenic variants and those without pathogenic variants by Fisher’s Exact test.

### Statistical analysis

All statistical analysis was performed by R version 3.6.1. Student’s t-test was used for pairwise numeric vector comparison, realized by *t* test in *R*. Chi-square test (*λ*2.test) was used for allele frequency comparison. Multiple-test was adjusted by “bonferroni” strategy.

## Supplementary Information

**Additional file 1.** Manually curated CFTR variants’ pathogenicity.

**Additional file 2.** Containing supplementary figures 1–6, supplementary table 3, supplementary table 4, and supplementary notes.

**Additional file 3.** Collected variants list of Caucasian-specific CF screening panels.

**Additional file 4.** Designed capture regions of CES and WES on *CFTR* gene.

## Data Availability

The data that support the findings of this study are either included in the article (or in its supplementary files) or available from the corresponding author on reasonable request. The data are not publicly available due to privacy or ethical restrictions.

## Abbreviations

CF: Cystic fibrosis
CFTR: CF transmembrane conductance regulator
CCGT: Chinese Children's Rare Disease Genetic Testing Clinical Collaboration System
AF: Allele frequency
MAF: Minor allele frequency
NFE: Non-Finland European population
FIN: Finnish in Finland
AMR: Admixed American population
SAS: South Asian
EAS: East Asian population
SNP: Single nucleotide polymorphisms
NBD: Nucleotide-binding domain
TMD: Transmembrane domain
WES: Whole exome sequencing
CES: Clinical exome sequencing
WGS: Whole genome sequencing
OR: Odds ratio
P/LP: Pathogenic/likely pathogenic
VUS: Variant of unknown significance
