# Supplementary material for: Systematic estimation of cystic fibrosis prevalence in Chinese and genetic spectrum comparison to Caucasians
Source: Orphanet J Rare Dis. 2022 Mar 21;17:129. doi: 10.1186/s13023-022-02279-9 (PMC8935702; doi:10.1186/s13023-022-02279-9)
Supplement: Supplementary file 2 — Additional file 2. Containing supplementary figures 1–6, supplementary table 3, supplementary table 4, and supplementary notes. [file 13023_2022_2279_MOESM2_ESM.docx]

**Supplementary Figures**


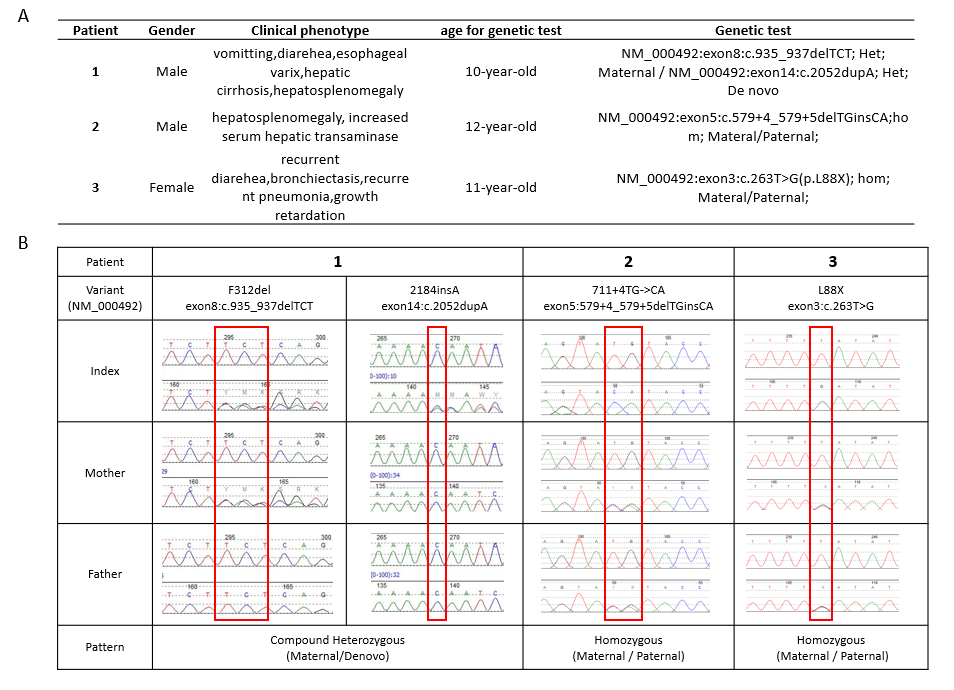


**Supplementary Figure 1. Clinical and genetic information of three patients with CF genetic identification.**

The basic clinical information and detailed genetic test results of three CF patients were listed. The Sanger results within family members were showed in the table.


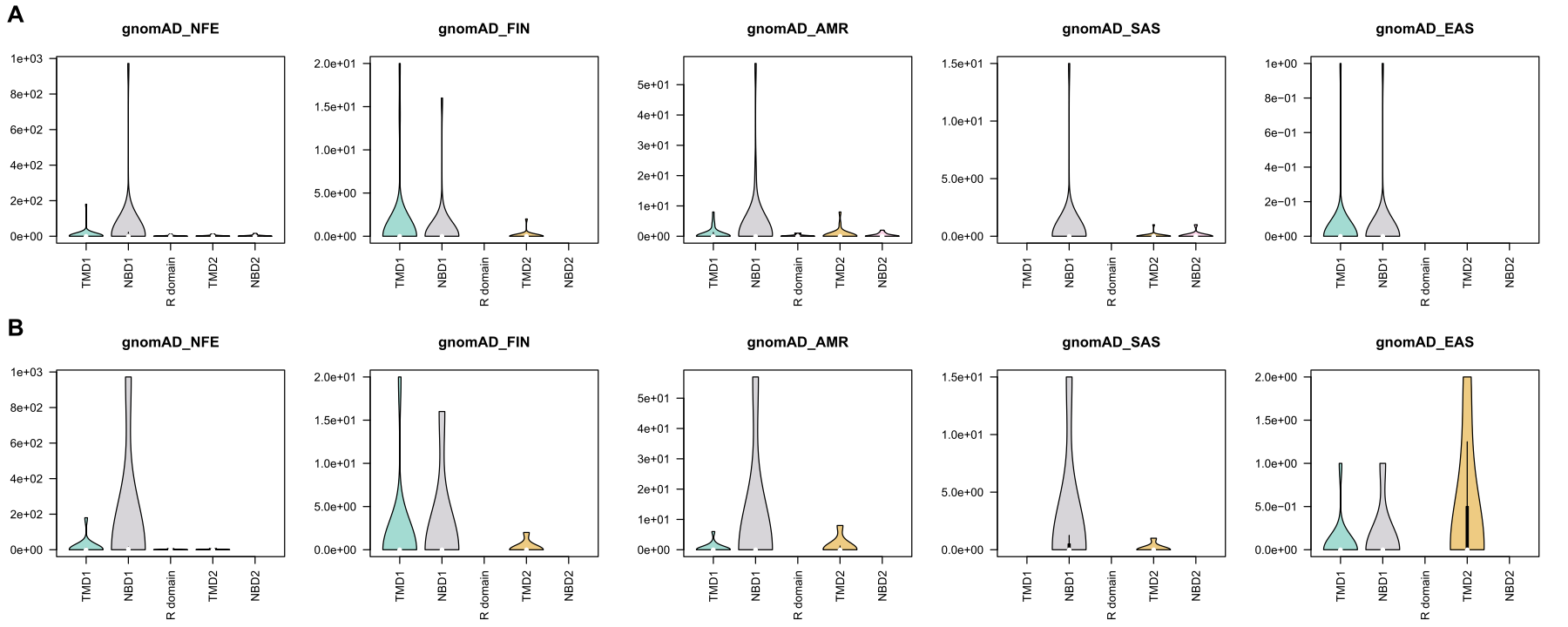


**Supplementary Figure 2. The total allele frequency of pathogenic variants of different population in each CFTR domain.**

Variants in Caucasian-specific panels (A) and Chinese-specific panels (B) are mapped to CFTR protein domains.


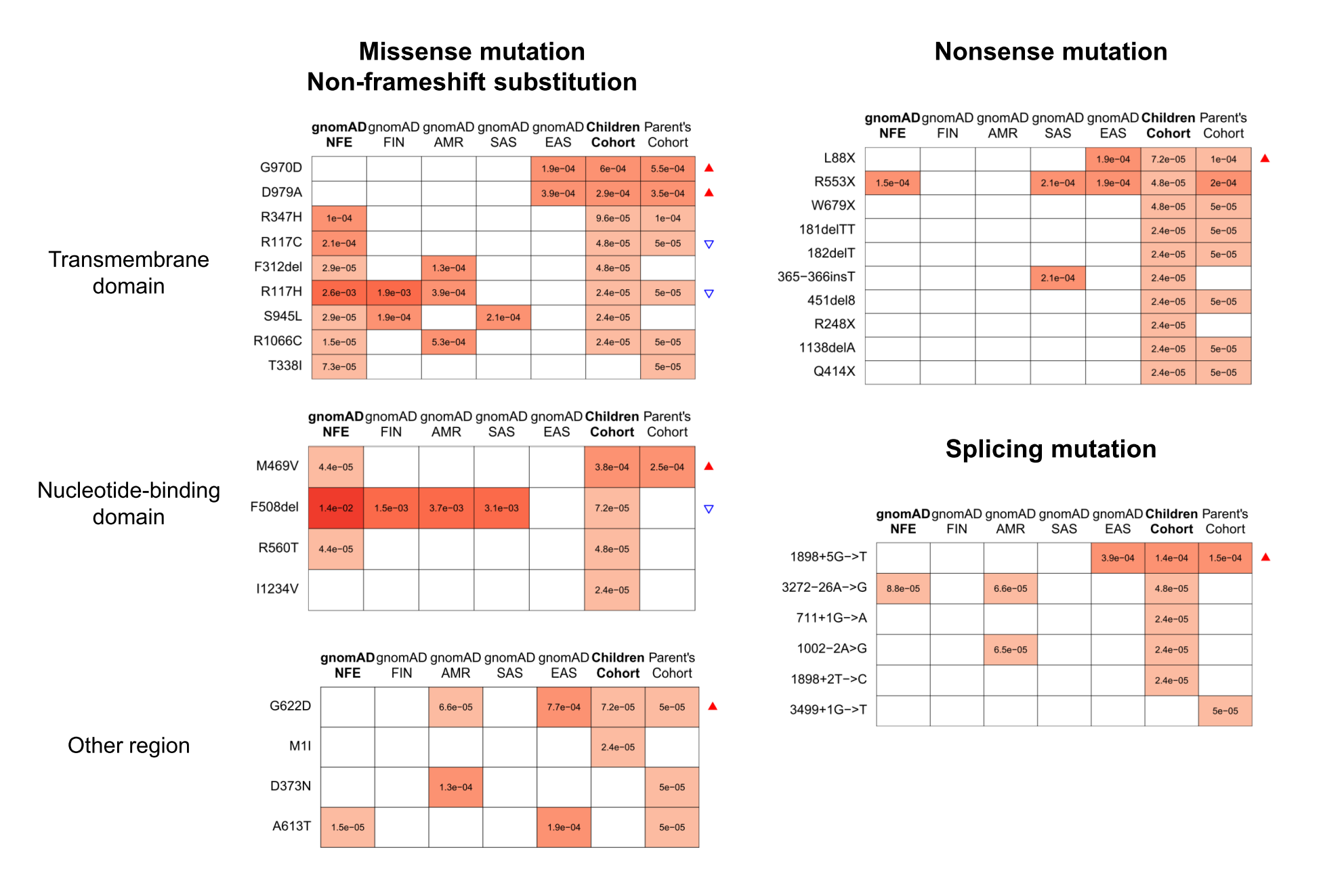


**Supplementary Figure 3. Allele frequency comparison for top 10 pathogenic variants for the three mutation types.**

The value in each box shows the AF for each variant (row) in each population (column). The blank box means the variant has not been detected in the population. All variants will be show if there are less than 10 variants in the mutation type. The red triangle highlights the six variants with significantly higher AF in our children patient cohort compared with gnomAD-NFE, and the blue triangle for the three significantly lower AF variants (all P-value<0.1).


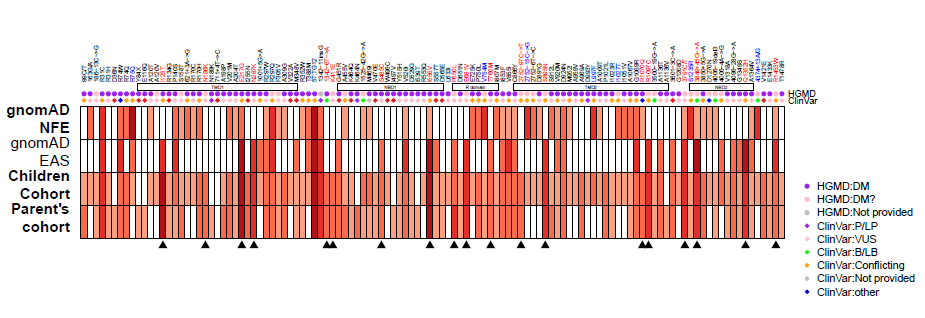


**Supplementary Figure 4. Allele frequency comparison for conflicting non-pathogenic variants.**

The color in each box shows the AF for each variant (column) in each population (row). Variants color in red (also bottom with black triangle) represent higher AF in children and parent’s cohorts compared with gnomAD-NFE (P-value<0.01 and OR>10), and blue vice versa (P-value<0.01 and OR<0.1). The lines with circles show the reported-level for each variant in HGMD and diamonds for ClinVar database. The five rectangles indicate the protein domains where the variants located in.


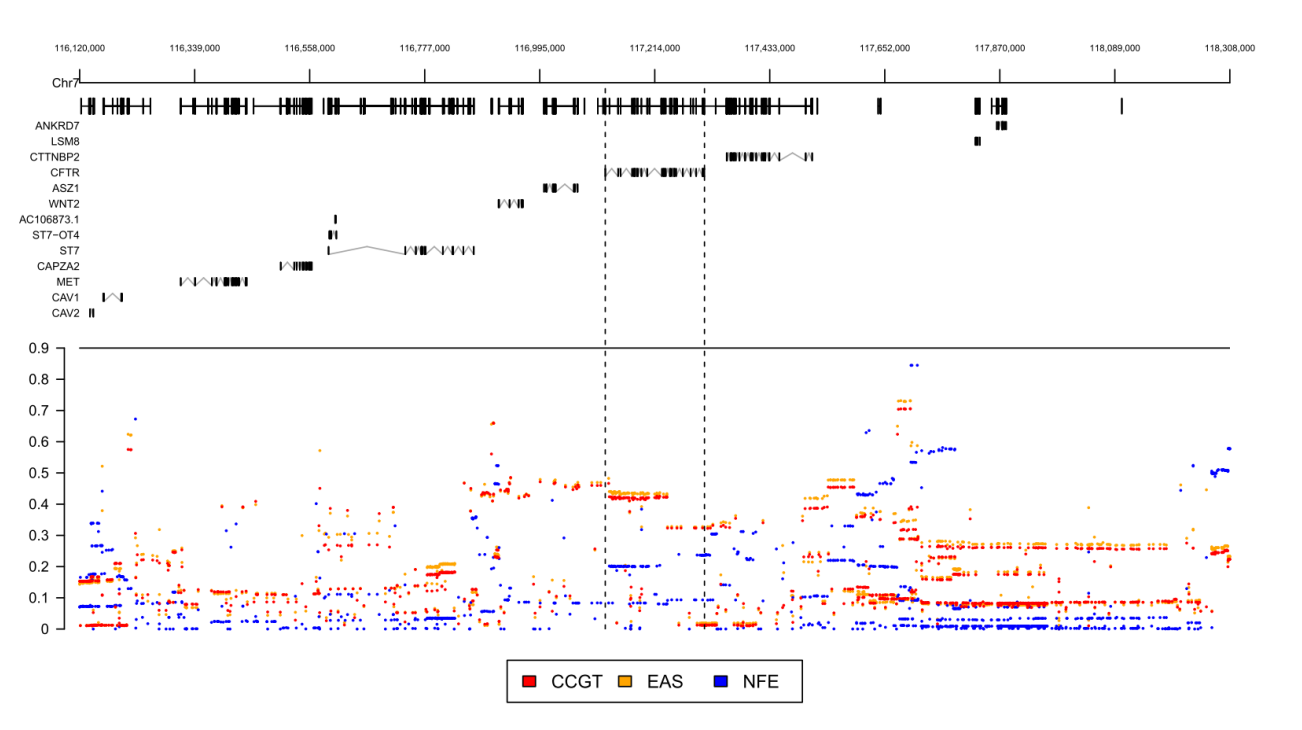


**Supplementary Figure 5. Allele frequency comparison for polymorphism sites around and on *CFTR*.**

We used another 2,067 CCGT WGS control-cohort for SNP allele frequency comparison. In total 3,916 SNPs (MAF>0.01) in *CFTR* ± 1Mb region were collected and 1,329 of them had significantly different MAF between CCGT-WGS cohort and gnomAD-NFE (all P<1e-30, OR>=2|OR<=0.5, MAF difference >0.05). Ninety SNPs were located in *CFTR* gene region. Seventy-eight intron-SNPs had higher MAF in CCGT WGS cohort, while ten intron-SNPs and two linked exon-SNPs, 4389G/A (rs1800136, c.4389G>A, AF=0.235) and chr7:117308413:C>T (rs1042180, c.*1251C>T, AF=0.013), had significantly higher MAF in gnomAD-NFE.


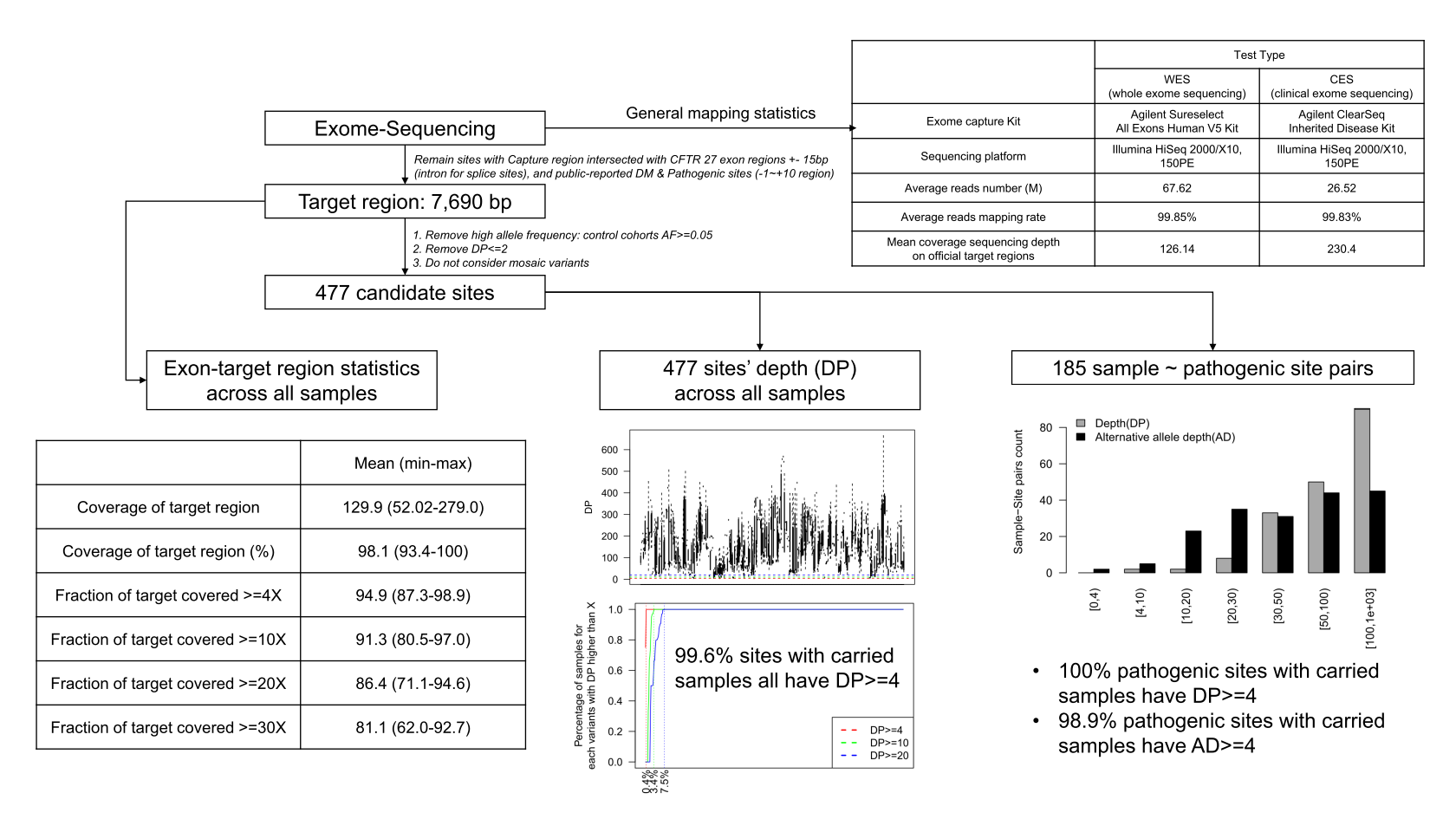


**Supplementary Figure 6. Quality control of next generation sequencing for *CFTR* gene.**

The work flow showed the methods and results of quality control (QC) of whole exome sequencing and clinical exome sequencing data used for CF prevalence estimation.

**Supplementary Tables**

**Supplementary Table 1**. Manually curated *CFTR* variants’ pathogenicity.

**Supplementary Table 2**. Collected variants list of Caucasian-specific CF screening panels

**Supplementary Table 3**. Estimated CF affected frequency by two screening panels with Bayesian framework method in different populations.

|  | **Caucasian−specific CF Panel** | **Chinese−specific CF panel** |
| --- | --- | --- |
| **gnomAD NFE** | 1/1663 (1/1832~1/1516) | 1/3124 (1/3501~1/2802) |
| **gnomAD FIN** | 1/33036 (1/58195~1/20755) | 1/76282 (1/156141~1/43510 |
| **gnomAD AMR** | 1/9548 (1/13302~1/7117) | 1/37952 (1/61445~1/25278) |
| **gnomAD SAS** | 1/15797 (1/32287~1/9021) | 1/55741 (1/156548~1/26635) |
| **gnomAD EAS** | 1/376784 (1/2271312~1/130628) | 1/173900 (1/704548~1/70107) |
| **CCGT Children cohort** | 1/1387395(1/2940462~1/774605) | 1/143171(1/213769~1/101160) |
| **CCGT Parent’s cohort** | 1/872437(1/2384062~1/422626) | 1/110127(1/192111~1/69638) |

**Supplementary Table 4**. Allele frequency on different haplotypes of rs213950-rs104200 in cohorts with different condition.

| **Cohort Group** | **rs213950-rs1042077** | **G-T**  **(AF)** | **G-G**  **(AF)** | **A-T**  **(AF)** | **A-G**  **(AF)** |
| --- | --- | --- | --- | --- | --- |
| **Alleles with Pathogenic Sites**  **(N: number of alleles)** | F508del homozygous  from published population (Vecchio-Pagán et al., 2016)  (N=762*2) | 0 | 0 | **0.997** | 0.003 |
|  | F508del carrier alleles  from CCGT Exome Children cohort  (N=3) | 0 | 0 | 0.333 | 0.667 |
|  | F508del carrier alleles  from CCGT Exome Parent’s cohort  (N=0) | 0 | 0 | 0 | 0 |
|  | G970D carrier alleles  from CCGT Exome Children cohort  (N=25) | 0 | 0.040 | 0 | **0.960** |
|  | G970D carrier alleles  from CCGT Exome Parent’s cohort  (N=11) | 0 | 0 | 0.090 | **0.910** |
| **Control Cohorts**  **(N: number of alleles)** | 1000 Genome NFE cohort  (N=390*2) | **0.628** | 0.008 | 0.095 | 0.269 |
|  | CCGT Exome  Children cohort  (N=20,905*2) | **0.553** | 0.013 | 0.009 | 0.425 |
|  | CCGT Exome  Parent’s cohort  (N=10,038*2) | **0.557** | 0.014 | 0.010 | 0.419 |
|  | CCGT WGS cohort  (N=2,067*2) | **0.563** | 0.013 | 0.010 | 0.414 |
|  | 1000 Genome EAS cohort  (N=596*2) | **0.624** | 0.008 | 0.003 | 0.364 |

**Supplementary Table 5**. Designed capture regions of CES and WES on *CFTR* gene.

**Supplementary Notes**

**Affected frequency estimation**

Initially, we collected a children cohort and a parent’s cohort with 20,909 and 10,042 individuals, respectively. After curation of CFTR pathogenic variants, we retrospectively identified three CF patients and one suspect CF patient. Two CF patients were conducted the genetic tests with their parents. To construct a relative healthy population for CF, we exclude the four children and four parents from the two cohorts, resulting in 20,905 children and 10,038 parents.

Take parent individual cohort as example. The total number of individuals is n=10,038 (5012 males and 5026 females), thus the total number of alleles is m=n*2. The total number of individuals carry P/LP CFTR variants in this cohort is x=60 (38 males and 22 females).

***Strategy I: carrier frequency***

The carrier frequency is: $\frac{x}{n}=1/167$

The couple’s carrier risk is: ${(\frac{x}{n})}^{2}=1/27989$

As CFTR is AR-inherited. The risk for CF child is: $\frac{1}{4}*\left( \frac{x}{n} \right)^{2}=1/111957$

***Strategy II: permutation-and-combination***

The hypothesis is to calculate the probability of affected child by random choosing a male individual and a female individual:

$$\frac{1}{4}*\frac{C_{38}^{1}*C_{22}^{1}}{C_{5012}^{1}*C_{5026}^{1}}=\frac{1}{120528}$$

***Strategy III: Bayesian framework***

According to *Schrodi et al*(Schrodi et al., 2015), the calculation process is as following.

Assume the affected allele probability is *q,* thus the CF affected probability is $q^{2}$ as CF is autosomal recessive disease.

$$P\left( q | X=x;m=2n \right)=\frac{P(X|q)P(q)}{\int_{0}^{1} P(X|q)P(q)dq}$$

The likelihood distribution is Binomial distribution:

$$P\left( X=x;m=2n \right)=\left( \begin{matrix} 2n \\ x \end{matrix} \right)p^{x}{(1-p)}^{2n-x}$$

In Bayesian probability theory, if the posterior distributions p(θ|x) are in the same family as the prior probability distribution p(θ), the prior and posterior are then called conjugate distributions, and the prior is called a conjugate prior for the likelihood (ref: <https://en.wikipedia.org/wiki/Conjugate_prior>). In the condition that the likelihood distribution is Binomial distribution and if the prior probability distribution follows Beta distribution ($\alpha=v,\beta=w$), the posterior distribution is also the Beta distribution ($\alpha=v+x,\beta=w+2n-x$). If $x\gg v$ and $2n-x\gg w$, we could neglect $v$ and $w$.

We could estimate the posterior mean value of $q^{2}$ as the second moment of posterior Beta distribution ($\alpha=x,\beta=2n-x$):

$${(\frac{\alpha}{\alpha+\beta})}^{2}+\frac{\alpha\beta}{\left( \alpha+\beta\right)^{2}\left( \alpha+\beta+1 \right)}$$

In the parent individual cohort, $\alpha=60,\beta=20016$ and the estimated prevalence is 1/110,127.

**References**

Schrodi, S.J., DeBarber, A., He, M., Ye, Z., Peissig, P., Van Wormer, J.J., Haws, R., Brilliant, M.H., Steiner, R.D., 2015. Prevalence estimation for monogenic autosomal recessive diseases using population-based genetic data. Human genetics 134(6), 659-669.

Vecchio-Pagán, B., Blackman, S.M., Lee, M., Atalar, M., Pellicore, M.J., Pace, R.G., Franca, A.L., Raraigh, K.S., Sharma, N., Knowles, M.R., 2016. Deep resequencing of CFTR in 762 F508del homozygotes reveals clusters of non-coding variants associated with cystic fibrosis disease traits. Human genome variation 3(1), 1-9.
